# Dataset of plant community composition in the Zumwalt Prairie Preserve, Oregon, USA

**DOI:** 10.1016/j.dib.2019.104690

**Published:** 2019-10-22

**Authors:** Bryan A. Endress, Joshua P. Averett

**Affiliations:** a Eastern Oregon Agriculture Research Center - Union Station, Oregon State University, Union, OR, 97883, USA; b Eastern Oregon Agriculture and Natural Resource Program, Department of Animal and Rangeland Sciences, Oregon State University, One University Blvd, La Grande, OR, 97850, USA

**Keywords:** Invasive species, *Ventenata dubia*, Grassland conservation, Plant community ecology

## Abstract

These data support the research article: “Non-native species threaten the biotic integrity of the largest remnant Pacific Northwest Bunchgrass prairie in the United States” Endress et al. (2019) [1].The data were collected at the Zumwalt Prairie Preserve (Zumwalt), northeastern Oregon, USA, and include vascular plant species abundance matrices from 123 plots sampled in 2008 and 2009 and the estimated abundance of dominant species in community space.

**Table:** Specifications table

Subject area	Agricultural and Biological Sciences (General)
More specific subject area	Grassland vegetation community ecology
Type of data	Map, Figure, Table (csv files)
How data was acquired	Field sampling within permanent plots
Data format	Raw, Analyzed
Parameters for data collection	Field collection of all vascular plant species in 2008 and 2009
Description of data collection	Navigated to plots via GPS; Line-point intercept sampling every 1 m along 3 50-m transects; identification of all vascular plant species.
Data source location	Zumwalt Prairie Preserve, Oregon, USA. (45˚58′ N, 116° 97′ W)
Data accessibility	With the article
Related research article	B.A. Endress, J.P. Averett, B.J. Naylor, L.R. Morris, R.V. Taylor. 2019. Non-native species threaten the biotic integrity of the largest protected remnant Pacific Northwest Bunchgrass prairie in the United States. Applied Vegetation Science.

**Table:** 

**Value of the data** • **Why are these data useful?** These data provides plant community information for one of the largest remaining Pacific Northwest Bunchgrass grasslands, an endangered and one of the least studied major vegetation types in the world. • **What is the additional value of these data?** The data presented includes community patterns coincident with invasion by a sparsely studied non-native annual grass, *Ventenata dubia.* • **How can these data be used for further insights?** Future repeated measurements can be compared to this data to reveal long-term vegetation community dynamics.

## 1 Data

**Fig. 1:**
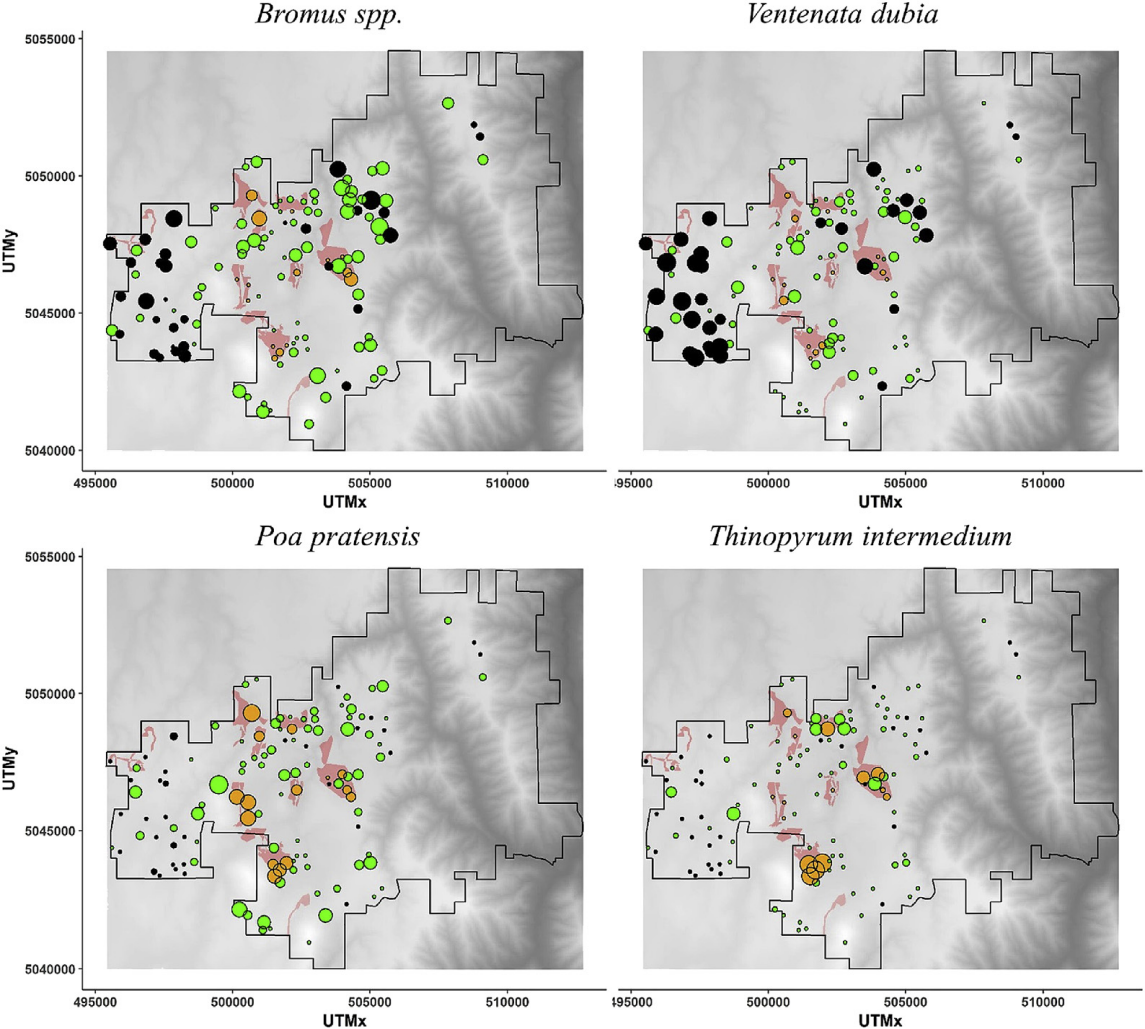
Distribution and abundance of the four most abundant non-native species across within Zumwalt Prairie Preserve sampled in between 2008 and 2009. The center of the circle indicates the location of the plot; the size of the circle reflects the abundance; the color indicates the plant community (orange = old fields; green = mesic prairie; black = xeric prairie). The light red polygons indicate locations of old fields.

**Fig. 2:**
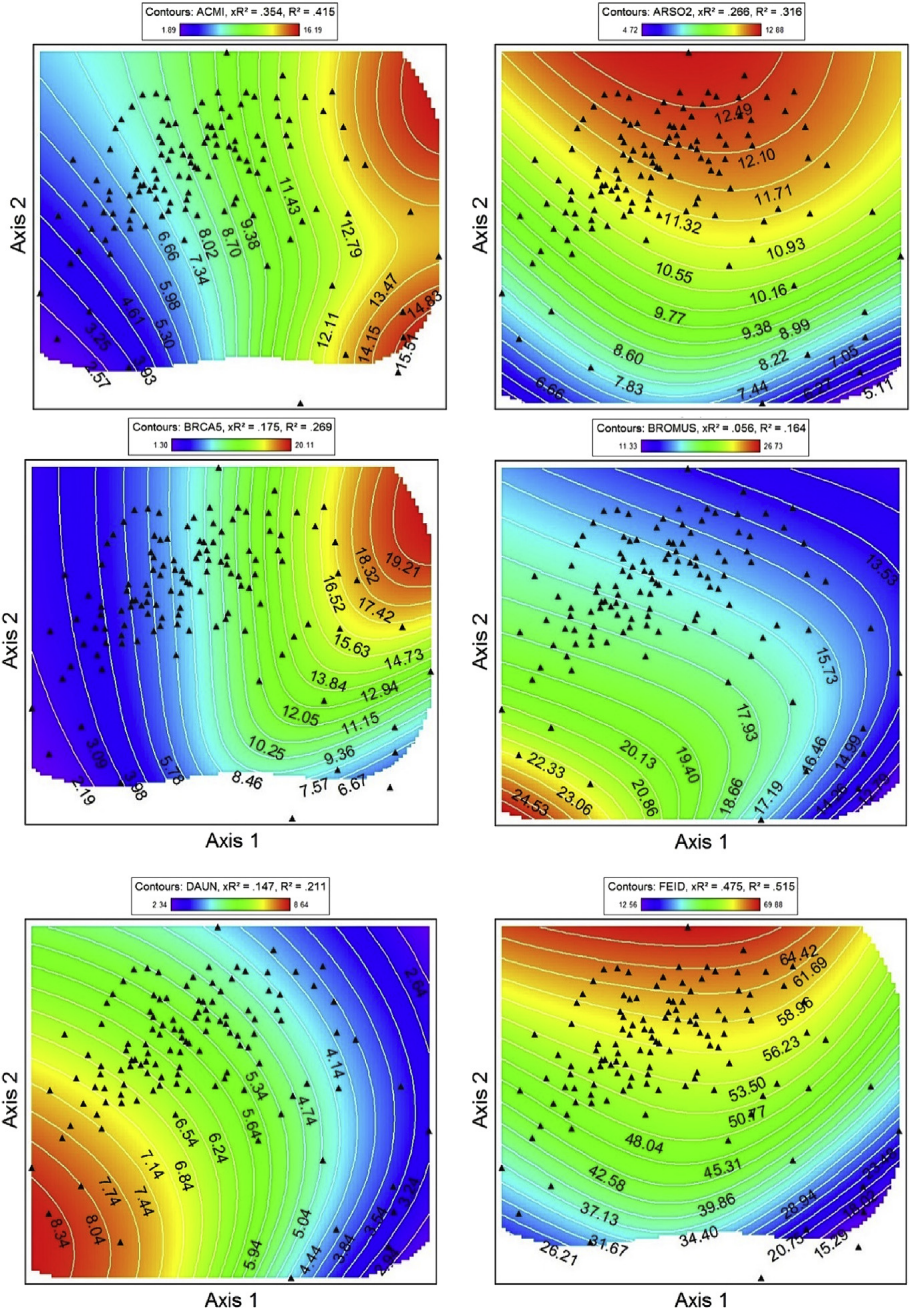

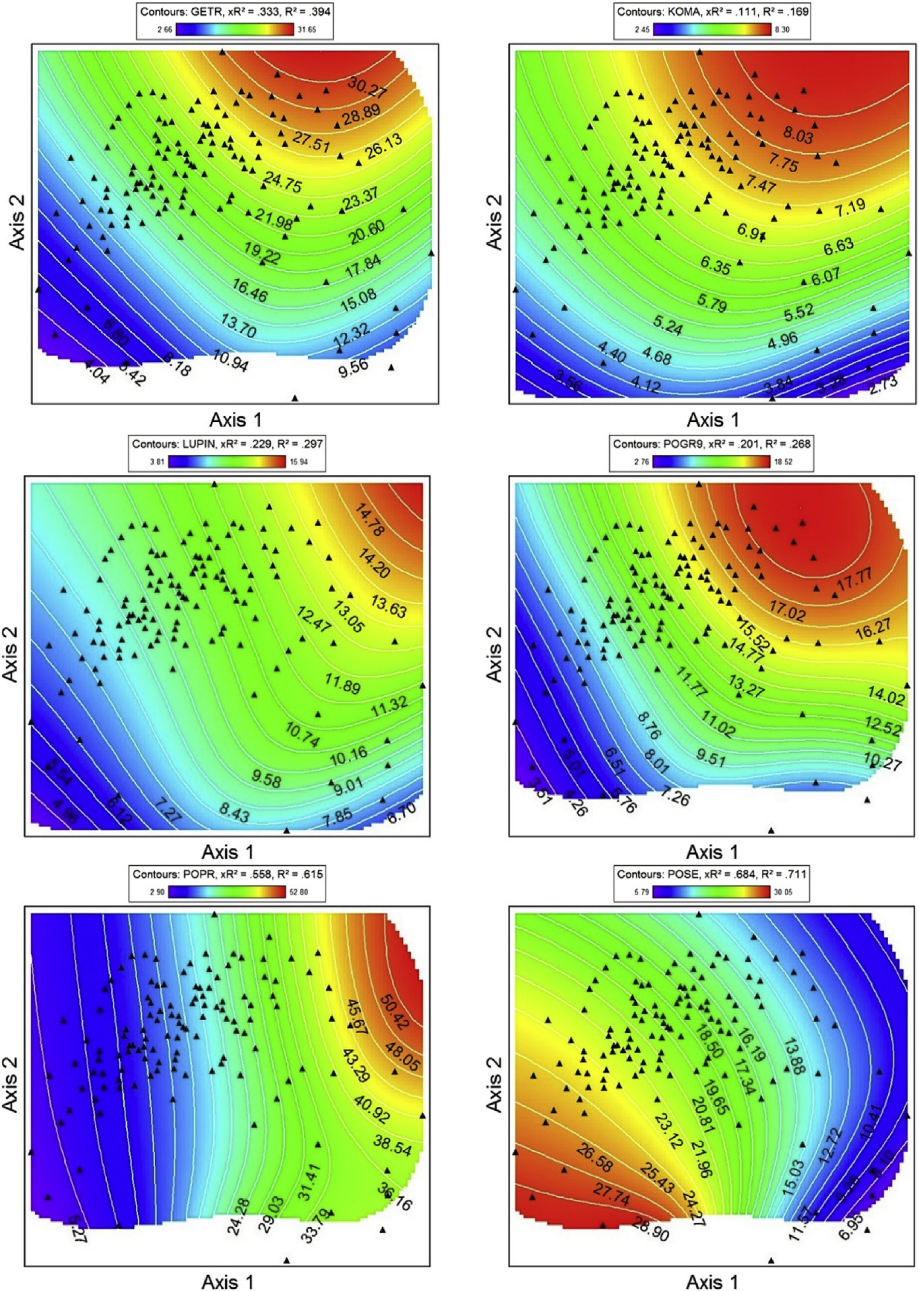

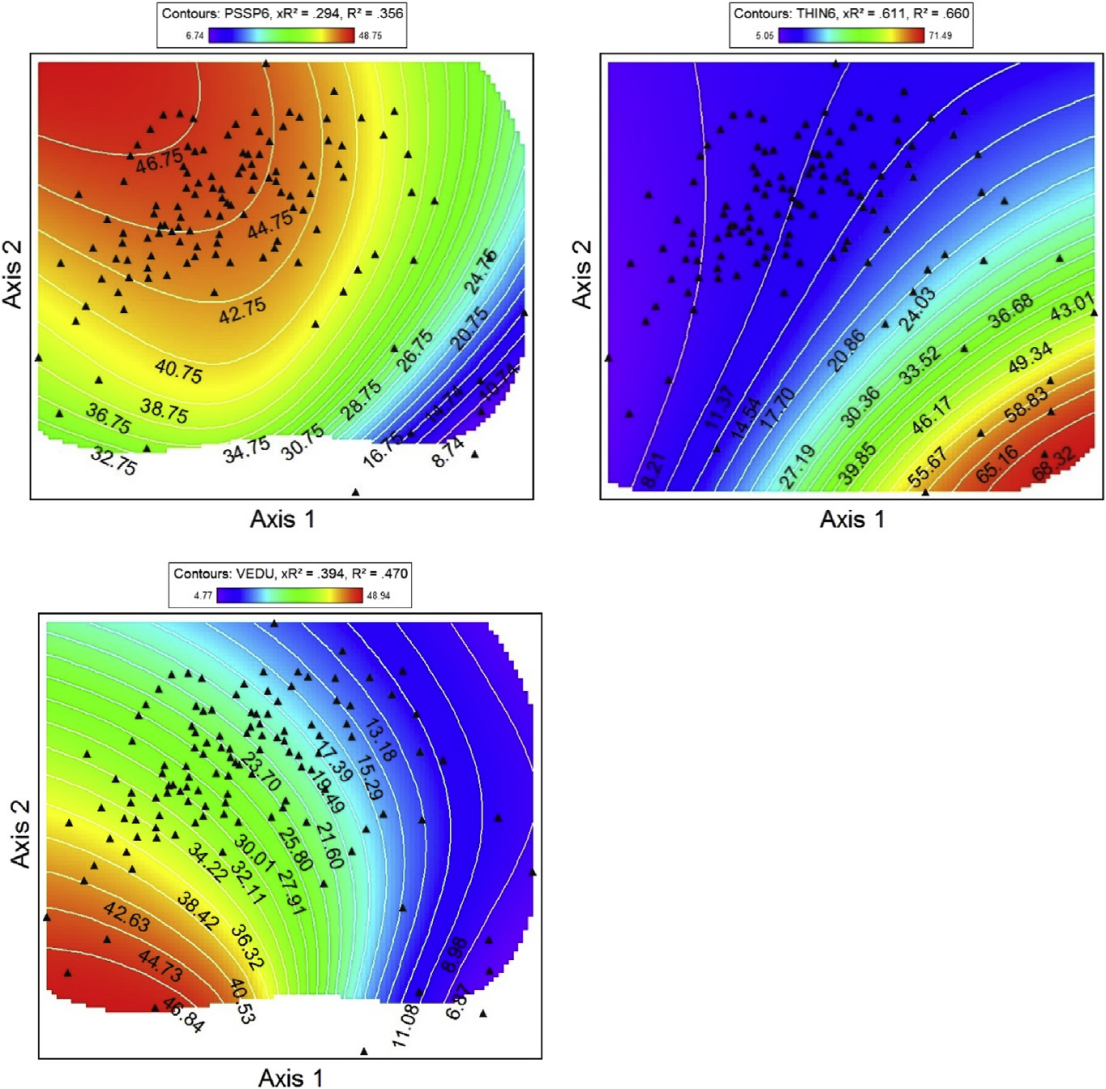
NPMR generated contour plots showing species abundance (% foliar cover) in 2008 & 2009 as a function of NMS ordination axes. ACMI = *Achillea millefolium*, ARSO2 = *Arnica sororia*, BRCA5 = *Bromus carinatus*, Bromus = *Bromus arvensis* & *Bromus hordeaceaus*, DAUN = *Danthonia unispicata*, FEID = *Festuca idahoensis*. Red corresponds to high foliar cover, and blue indicates lower cover. NPMR generated contour plots showing species abundance in 2008 & 2009 as a function of NMS ordination axes. Red corresponds to high foliar cover, and blue indicates lower cover. GETR = *Geum triflorum*, KOMA = *Koeleria macrantha*, Lupin = *Lupinus* spp., POGR9 = *Potentilla gracilis*, POPR = *Poa pratensis*, POSE = *Poa secunda*. NPMR generated contour plots showing species abundance in 2008 & 2009 as a function of NMS ordination axes. Add species codes. Red corresponds to high foliar cover, and blue indicates lower cover. PSSP6 = *Pseudoroegneria spicata*, THIN6 = *Thinopyrum intermedium*, VEDU = *Ventenata dubia*. Red corresponds to high foliar cover, and blue indicates lower cover.

The Pacific Northwest Bunchgrass Prairie (PNB) ecosystem is one of the most endangered and among the least studied grasslands in North America [[2], [3]]. These data were obtained by sampling vascular plant composition across the Zumwalt Prairie Preserve (Zumwalt; northeastern Oregon), the largest intact remnant of PNB in the United States. Sampling occurred in 2008 & 2009. The presented data include: (1) A map (Fig. 1) showing the distribution and abundance of the four most abundant non-native species across within Zumwalt Prairie Preserve sampled in between 2008 and 2009; (2) Regression (NPMR) generated contour plots (Fig. 2) of species foliar cover in community space, community space being defined by the two primary axes generated using Non-metric Multidimensional Scaling (NMS); and (3) Downloadable CSV files (Appendix A and Appendix B) that include vascular plant species abundance (foliar cover) summaries, and relationships to community variation across the study area as well as raw species abundance matrices by plot. Refer to Ref. [1] for detailed interpretation, discussion, and related analyses.

## 2 Experimental design, materials, and methods

131 grassland plots were established using a stratified random sampling design. The Zumwalt was categorized into prairie and canyon lands; the canyon lands were excluded from the study area. The remaining prairie was divided into quarter-quarter (0.25 × 0.25 miles or 16.2 ha) sections based on the US Public Land Survey System, and plots were randomly located within each quarter-quarter section [1]. A GEO-Explorer Trimble 3 handheld Global Positioning System was used to navigate to the selected plots. Three line-point intercept [4] transects oriented in a spoke design and radiating out from the center of the plot at 0°, 120°, and 240° relative to magnetic North were established within each plot [5]. Species intercepts with transects were observed at 1 m increments, for a total of 150 points sampled (50 per transect) in each plot. Percent foliar cover (per plot) was calculated as the total number of hits for a given species divided by the 150 total possible points multiplied by 100. Because multiple species, at different canopy layers, are often intercepted at the same point, total plot cover can be >100%. Presence absence of dominant non-native vascular plant species were also recorded within subplots (0.4 × 0.4 m) spaced at 5 m increments along each transect line for a total possible frequency of 30 subplots per plot. Eight plots were excluded from analyses because they had burned within three years prior to sampling. Therefore, data from 123 plots was used in the analysis.

To evaluate spatial patterns of non-native species abundance and their relationships to community composition and land use, the foliar cover of dominant species (native and non-native) and the location of old fields were plotted spatially as bubble maps across the Zumwalt study area using the ggplot2 package in R [6,7]. Perennial non-native grass species were concentrated in or adjacent to old fields, while annual non-natives were more widely distributed, with higher abundances in uncultivated areas particularly those with more xeric conditions (Fig. 1).

Non-metric multidimensional scaling (NMS [8]) was used to extract the dominant species composition gradients in our dataset [1]. Three-dimensional response surfaces of species abundance (foliar cover) in NMS ordination space (Fig. 2) were generated for dominant native and non-native species using Non-parametric Multiplicative Regression [9] with a local mean estimator, Gaussian kernel smoother, and automatic average minimum neighborhood size option in PC-ORD 7.0 [8]. NPMR automatically models interactions among predictors and has built in over-fitting protection consisting of a leave-one-out cross validation method during model fitting [9]. Cross validated *R*^2^ (_X_R^2^) and *R*^2^ values were both used to evaluate model fits. Cross validated *R*^2^ values differ from the conventional *R*^2^ because it is based on the exclusion of each data point from the estimate of the response at that point [9].

## Appendix A Supplementary data

Multimedia component 1 Supplementary file: “Zumwalt_Supplemetary_Info.csv”; columns: Species (species code; USDA Plant Database); Scientific name; Common Name; Family; Form (F, forb; G; grass; Shrub); Life Span (P, Perennial; A, Annual); Origin (N, Native to contiguous United States; NN, non-native); Rhizomatous (Yes/No); Rank Abundance (based on foliar cover); Total Foliar Cover (sum of foliar cover); Rank Frequency (plot level frequency); Frequency (plot level); Proportion of Plots Occupied; Mean Foliar Cover (%; all plots); Foliar Cover Standard Deviation; Axis 1 r (correlation coefficient for species foliar cover plotted against NMS ordination axis 1); Axis 1 r2 (linear coefficient of determination for species foliar cover plotted against NMS ordination axis 1; the + / - indicated the direction of the correlation); Axis 2 r (correlation coefficient for species foliar cover plotted against NMS ordination axis 2); Axis 2 r2 (linear coefficient of determination for species foliar cover plotted against NMS ordination Axis 2; the + / - indicated the direction of the correlation); xR2(NPMR)(cross-validated r-squared value for the best 2 predictor model with response = species foliar cover and predictors = NMS axes 1 & 2); NMS (Y, species was included in the NMS analyses; N, species was excluded from the NMS analyses). Multimedia component 1

Multimedia component 2 Supplementary file: “Zumwalt_Spp_Matrix.csv” is the species matrix and includes sample units (plots) as rows, species as columns, and each cell represents the foliar cover estimate (percent) for that species in a given plot. Multimedia component 2
